# Does musculoskeletal pain interfere with motor learning in a gait adaptation task? A proof-of-concept study

**DOI:** 10.1186/s12891-022-05237-5

**Published:** 2022-03-23

**Authors:** Frédérique Dupuis, Benoit Pairot de Fontenay, Jason Bouffard, Marc Bouchard, Laurent J. Bouyer, Catherine Mercier, Jean-Sébastien Roy

**Affiliations:** 1 Centre interdisciplinaire de recherche en réadaptation et intégration sociale (Cirris), Centres intégrés universitaires de santé et de services sociaux de la Capitale Nationale (CIUSSS-CN), Quebec City, Canada; 2 Départment of Rehabilitation, Université Laval, Quebec City, Canada; 3 Centre Hospitalier Universitaire de Québec, Quebec City, Canada

## Abstract

**Background:**

Experimental pain during gait has been shown to interfere with learning a new locomotor task. However, very few studies have investigated the impact of clinical pain on motor learning due to the challenges associated with clinical populations.

**Objective:**

The first objective of this proof-of-concept study was to determine the feasibility to obtain two groups of participants with chronic ankle pathology with or without residual pain while walking. The second objective was to evaluate the impact of clinical musculoskeletal pain on motor learning during gait.

**Methods:**

Participants with chronic isolated ankle pathology were recruited and their personal and clinical characteristics were collected (functional performance, dorsiflexion maximal strength, range of motion). To assess motor acquisition (Day 1) and retention (Day 2), participants performed an adaptation task on two consecutive days that consisted of walking while experiencing a perturbing force applied to the ankle. The level of pain during the task was measured, and participants who reported pain were attributed to the Pain group and participants without pain to the No Pain group. Learning performance was assessed by measuring ankle kinematics (Mean plantarflexion absolute error) and learning strategy was assessed by measuring the Relative timing of error and the tibialis anterior (TA) electromyographic activity.

**Results:**

Twenty-five participants took part in the experiment. Eight (32%) were excluded because they could not be included in either the Pain or No Pain group due to the intermittent pain, leaving eight participants in the Pain group and nine in the No Pain group. Both groups were similar in terms of baseline characteristics. Musculoskeletal pain had no influence on learning performance, but the learning strategy were different between the two groups. The No Pain group showed a TA activity reduction before perturbation between the days, while the Pain group did not.

**Conclusion:**

Some barriers were identified in studying musculoskeletal pain including the high rates of participants’ exclusion, leading to a small sample size. However, we showed that it is feasible to investigate clinical pain and motor learning. From the results of this study, musculoskeletal pain has no influence on motor learning performance but influences the learning strategy.

## Introduction

Pain is one of the most common and disabling symptoms following a musculoskeletal injury [1]. Pain is also the primary complaint when patients start their rehabilitation, and patients experiencing pain exhibit a poorer functional recovery than patients with similar injuries but without associated pain [2].

Although it is well accepted that pain interferes with motion, movement alterations observed in the presence of pain are often deemed to simply be a consequence of anticipating and minimizing pain [3]. However, basic research using experimental pain in humans have shown that the interactions between pain and movement is much more complex [4–9]. For instance, pain influences the excitability of the primary and secondary somatosensory cortex, the primary motor cortex and the spinal cord [6, 9], structures involved in motor learning. Using a force-field adaptation paradigm during walking, experimental muscle pain in ankle dorsiflexors has also been shown to alter the motor strategy used by healthy individuals without affecting their task performance [10]. Specifically, participants relied less on anticipatory motor strategies to adapt to the force perturbation when they experienced pain [10]. Interestingly, such motor strategy is retained when performing a transfer test the next day without pain. The presence of pain while walking could therefore interfere with the way we learn a new locomotor task after a lower limb musculoskeletal injury, and this may be associated with the poorer recovery reported after musculoskeletal injury.

To our knowledge, only a few studies have evaluated the effect of clinical pain on motor learning [11–14]. In the upper extremity, no impairment in learning skills were observed in people suffering from hand arthritis [11], fibromyalgia [12] or complex regional pain syndrome [13]. As for the lower limb, Rittig-Rasmussen et al. showed that participants with knee pain improved less their performance when training to a shoulder elevation tracking task than participants with neck pain [14]. There was, however, no pain-free control group for comparison which limits our ability to interpret these results [14]. To our knowledge, no study has evaluated the effects of clinical pain during a task relevant to populations with a lower limb injury such as locomotion, despite the fact that experimental pain applied to ankle dorsiflexors or around the ankle joint has been shown to impact on locomotor adaptation [10, 15, 16]. As a majority of patients treated for an isolated ankle injury still report symptoms 1 year after the injury and 61% of those patients still experience pain during walking 14 months after injury [1, 2, 17], it is important to better understand the impact of clinical pain on locomotor learning.

Up to now, all studies interested in locomotor learning have evaluated the effect of experimental pain on learning in healthy participants, but not in participant with musculoskeletal pain. While experimental pain models are useful tools to isolate the effects of pain on the variable under study, they are not entirely representative of pain experienced by people with musculoskeletal injuries [18]. Indeed, most experimental pain models have a very short-term effect, whereas chronic musculoskeletal pain develops over a long period, which might affect motor learning differently. However, there are many challenges associated with studying motor learning in clinical populations with musculoskeletal pain. For example, injury-related factors other than pain (e.g., stiffness, muscle atrophy and joint degeneration) might impact motor learning strategy. Additionally, it is difficult to know in advance whether and for which participants the task assessed will be painful [19], and if the above-mentioned injury-related cofactors will be balanced between groups of participants (pain vs no pain). Furthermore, the intensity of clinical pain is known to be highly variable from 1 day to another in a given participant [20], which generates a particular challenge in motor learning studies as skill acquisition and retention must be assessed on different days. Whether pain is stable or not across days appears to impact on retention based on experimental pain studies [15, 16]. All these methodological challenges probably explain why so few studies have focused on the effect of clinical musculoskeletal pain on motor learning so far [11–14], despite the clear clinical relevance of that question.

Therefore, the first objective of this proof-of-concept study was to determine the feasibility to obtain two groups of participants affected by chronic ankle pain with or without residual pain during walking, while otherwise presenting similar characteristics (e.g., in terms of age, anthropometric characteristics, type of injury, functional performance, strength and range of motion). The second objective was to explore the impact of chronic clinical musculoskeletal pain on motor acquisition and retention of a locomotor adaptation task by comparing participants affected by isolated ankle pathology with residual pain to participants also affected by isolated ankle pathology but without any pain during walking. We looked at global performance (quantity of movement error) and motor strategy (anticipatory or reactive muscle activations) while a force-field was repeatedly applied to perturb the gait pattern. The hypothesis was that participants with clinical musculoskeletal pain would use less anticipatory and more reactive strategies during the gait adaptation task than participants without pain, and that this would carry over to the retention phase [15].

## Methods

A convenience sample of 25 participants with isolated ankle pathology took part in the experiment. All participants were recruited through the orthopaedic department of a local hospital and the electronic mailing list of employees and students at *Université Laval*. The ethics committee of the *Centres intégrés universitaires de santé et de services sociaux de la Capitale Nationale* (CIUSSS-CN) (rehabilitation and social integration section) and of the *Centre hospitalier universitaire de Québec* granted the ethical approval. All participants provided their written informed consent.

Inclusion criteria were: 1) to be aged over 18; 2) to be living with one of these two isolated ankle pathologies: ankle fracture or ankle osteoarthrosis for at least 3 months; and 3) to be able to walk for at least 20 min without a walking aid. Exclusion criteria were: 1) to have a history of chronic pain or presence of pain unrelated to the ankle condition and 2) to have a neurological disorder that could affect task performance.

### Experiment

Baseline characteristics such as age, injury type and anthropometric characteristics were first collected on day one. Then, maximal ankle dorsiflexion strength (using a dynamometer) [21] and maximal weight-bearing dorsiflexion range of motion [22] were measured and participants filled three validated self-reported questionnaires: the Lower Extremity Functional Scale (LEFS), the Tegner Activity Scale (current level of activity) and the Pain Interference Subscale of the Brief Pain Inventory (BPI). The LEFS is a 20-item questionnaire assessing the impairment of the lower-extremity musculoskeletal system in everyday activities with a score ranging from 0 (minimal impairment) to 80 (maximal impairment) [23]. It has been validated in individuals with ankle pathologies [23]. The Tegner Activity Scale evaluates work and sports activities using a score ranging from 0 (maximal disability) to 10 (full participation in sports) [24, 25]. The BPI includes 11 items scored on a numeric 0 to 10 scale where 0 = no interference and 10 = total interference. The mean score on the 11 items was reported [24, 25]. The BPI was also completed on Day 2, to characterize the stability of pain experienced on Day 1 and Day 2 [26].

Thereafter, all participants performed a gait adaptation task on two consecutive days, to assess both motor acquisition (Day 1) and retention (Day 2). On each day, they walked on a treadmill at 1 m/s [27, 28] while wearing the robotized ankle foot orthosis (rAFO) on their injured side [29]. The rAFO is a custom-designed electrohydraulic ankle-foot orthosis that can produce several types of force fields during walking [29]. It has been used in several studies evaluating force-field adaptation paradigm during walking [16, 30]. Detailed information on the rAFO can be found in Noel et al. [29] During the gait task, the level of ankle pain was rated verbally every minute on a 0-10 numerical rating scale (0 = no pain and 10 = worst imaginable pain) and the mean level of pain during the task was reported. A familiarization period (5 min) preceded data collection.

### Motor learning test

Details of the experimental procedures have been previously described by Bouffard et al. [10, 15, 16] For 5 min, participants walked on the treadmill with the rAFO while no force field was applied to quantify baseline gait. For the next 5 min, the rAFO applied a force field resisting ankle dorsiflexion during the midswing phase of each stride (adaptation phase). The torque magnitude of the perturbation was constant during the entire adaptation phase. Participants were not told about the exact time at which the force field would be turned on. They were instructed to “overcome the perturbation in order to walk as normally as possible.” For the last 5 min, participants walked again without the force field to recover their normal walking pattern (washout). The rAFO actively cancelled torques produced by its mechanical components to minimise interference with gait pattern during baseline and washout periods (i.e. force cancellation mode) [31].

During the experiment, the ankle angle in the sagittal plane was recorded by an optical encoder located on the rAFO (encoder resolution is < 1°) [29]. The torque applied by the rAFO was measured by a load cell and the heel contact (custom made foot switch placed under the shoe) was recorded to calculate stride cycle duration. The tibialis anterior (TA) muscle activity was recorded on the trained lower limb using surface electromyography (EMG). The electrodes were placed just below the calf band of the rAFO, at 1/3 on the line between the tip of the fibula and the tip of the medial malleolus as recommended by the Surface Electromyography for the Non-Invasive of Muscles (SENIAM) guidelines [32].

### Variables of interest

Participants’ global performance (i.e., how much the participant can cancel the effect of the force field) was characterized using the Mean absolute ankle angle error. This measure represents the difference in the relative ankle angle curves between the baseline and the adaptation phases. The motor strategy used by the participant to overcome the force field during the adaptation phase was characterized by 1) the Relative timing of error (a measure of the temporal center of error distribution relative to the peak force command) [10] and by 2) the Tibialis anterior EMG activity change before and after the perturbation (peak force command [PFC]) (i.e., TA_ratioBeforePFC_ and TA_ratioAfterPFC_, indicators of feedforward and feedback control, respectively) [15].

Both the Mean absolute ankle angle error and the Relative timing of error were derived from generated error curves. Using the heel contact and rAFO control signals, data were separated into individual gait cycles and tagged as perturbed or non-perturbed strides. Ankle angle data were low-pass filtered with a second-order zero-lag Butterworth filter at 15 Hz. With the use of the ankle angle obtained from the optical encoder, the swing phase was identified as described by Bouffard et al. [16] and time normalized to 1000 points. A baseline swing phase ankle angle template was then created by averaging 45 of the last 50 baseline strides for each day (after removing the 5 most different strides from the mean). The ankle angle error was then calculated by subtracting point-by-point the baseline template values from each stride of the adaptation phase. The absolute value of ankle error of all 1000 swing phase points was averaged to define the Mean absolute error. An increased Mean absolute error represents a lower motor learning performance. In addition, changes leading to smaller (i.e., earlier) Relative timing of error during the adaptation phase represent switching to a more anticipatory strategy, while larger (i.e., later) values represent a more reactive strategy.

As for the Tibialis anterior EMG activity gains, EMG data were digitally filtered with a second-order zero-lag butterworth filter (bandpass 20–450 Hz) and rectified, and the envelope was extracted using a nine-point moving average [33]. As EMG activity precedes movement onset, the time window used for EMG analysis was extended by 30% of the identified swing phase, starting earlier to include the onset of TA stance-to-swing burst.

**Fig. 1:**
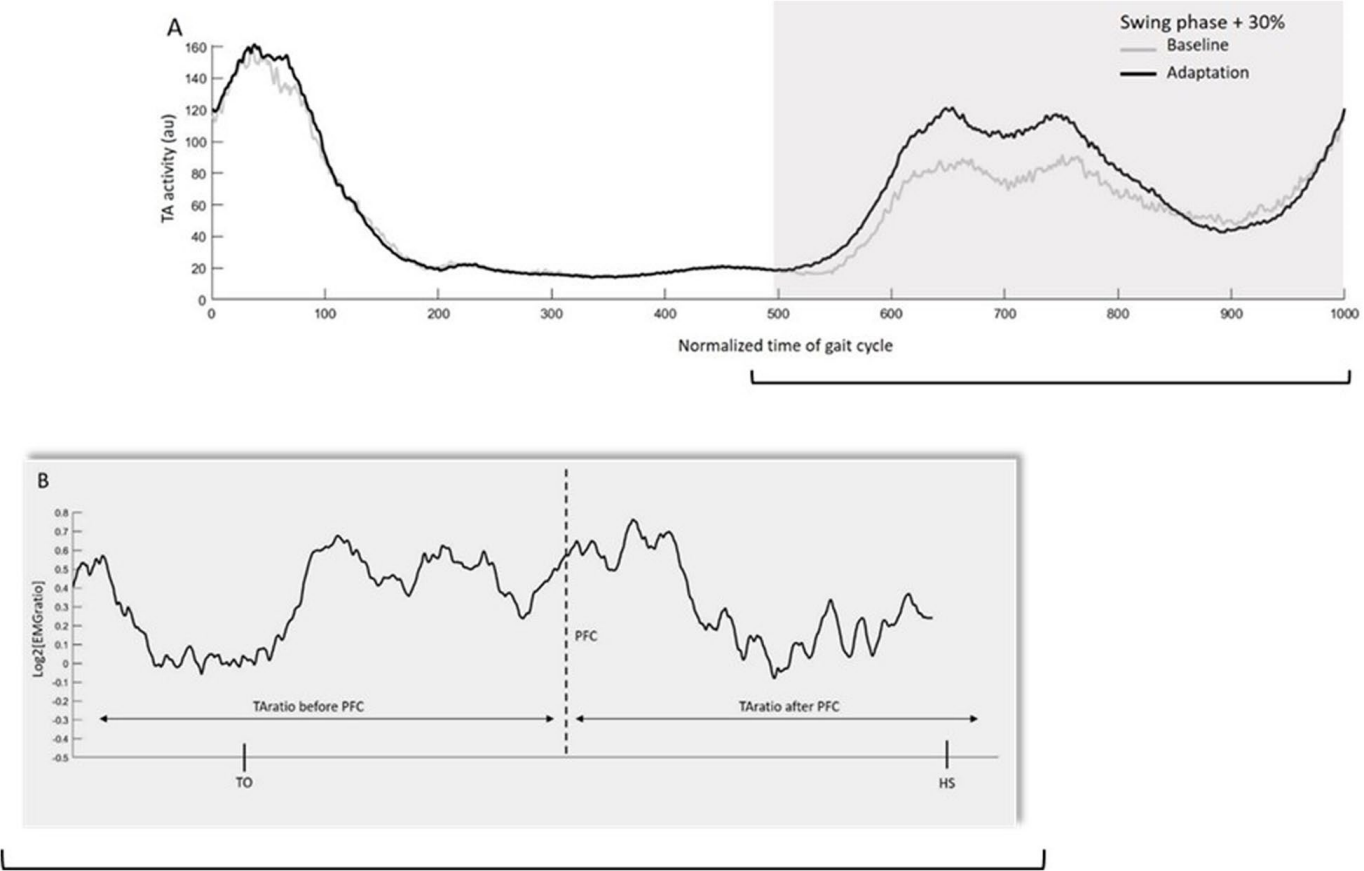
EMG analysis. **A** Tibialis anterior (TA) activity during baseline (gray trace) and adaptation (black trace). **B** point-by-point ratio of the early adaptation period’s TAratios. PFC, peak force command; EMG, electromyography; HS, heel strike; TO, toe off

To quantify changes in TA activity during adaptation, an EMG gain was calculated, consisting of a point-by-point ratio between the TA activity of adaptation divided by baseline (TAratio) (see Fig. 1 for an example). EMG gains were then linearized using a log2 transformation. Mean gains before (TA_ratioBeforePFC_) and after (TA_ratioAfterPFC_) PFC were computed. For more details on data analysis, see Bouffard et al. [10] All data were analysed using custom-made software written in MATLAB (The MathWorks Inc., Natick, USA).

### Statistical analysis

Participants who experienced pain constantly during the adaptation phase on both Day 1 and 2 were assigned to the Pain group (minimum pain at each time point ≥1/10). Participants who did not experience pain during the adaptation phase on both days were assigned to the No Pain group. Participants who had intermittent pain (e.g., ≥1/10 on Day 1 and < 1/10 on Day 2) were excluded from statistical analyses. Personal and clinical characteristics were compared between the Pain and No Pain groups using Mann Whiney tests and χ2 (e.g., age, gender, anthropometric characteristics, functional performance, strength, range of motion and pain during the task). The stability between the perceived levels of pain during the task on Day 1 and Day 2 was also evaluated by comparing participant’s scores between the days for the Pain group, using an Intraclass Coefficient Correlation (ICC; Two-way mixed effects) and a paired t-test [34]. The number (%) of participants excluded from analyses was reported as an indicator of protocol feasibility.

For the second aim, data from the Pain and No Pain groups were compared using a three-way non-parametric ANOVA for repeated measures (NparLD; Time [within subject]: Early vs. Late; Day [within subject]: Day 1 vs. Day 2 and Group [between subjects]: No Pain vs. Pain) on the following dependent variables: Mean absolute error, Relative timing of error and TAratios during the adaptation period. Time was characterized as Early adaptation (mean of strides 2–11 of the adaptation phase) and Late adaptation (mean of strides 151–200 of the adaptation phase). NparLD analyses are particularly relevant for small sample sizes and do not require normality of the data [35]. Effect sizes were reported as relative treatment effect (RTE). RTE is used to compare causal effect of a treatment on outcome; the distribution of the two groups is compared based on mean ranks and can thus be related to each other (≥.71 or ≤ .29: high effect; ≥.64 or ≤ .36: medium effect; ≥.56 or ≤ .44: low effect) [36].

Statistical analyses were conducted using the nparLD and AOV packages of the R software, respectively (version R.2.7.2.; R Foundation for Statistical Computing, Vienna, Austria). Mann Whiney test and χ2 were conducted in IBM SPSS Statistics (IBM SPSS Statistics 26, IBM Corp., NY, USA). Results are presented as means ± standard errors of the mean (SEM). Considering the exploratory design of this study and the statistical power limitation due to the small sample size, we decided to not apply correction for multiple comparisons for post hoc analyses. Level of statistical significance was set at *p* < 0.05.

## Results

### Feasibility of obtaining pain and no pain groups that are otherwise comparable

**Fig. 2:**
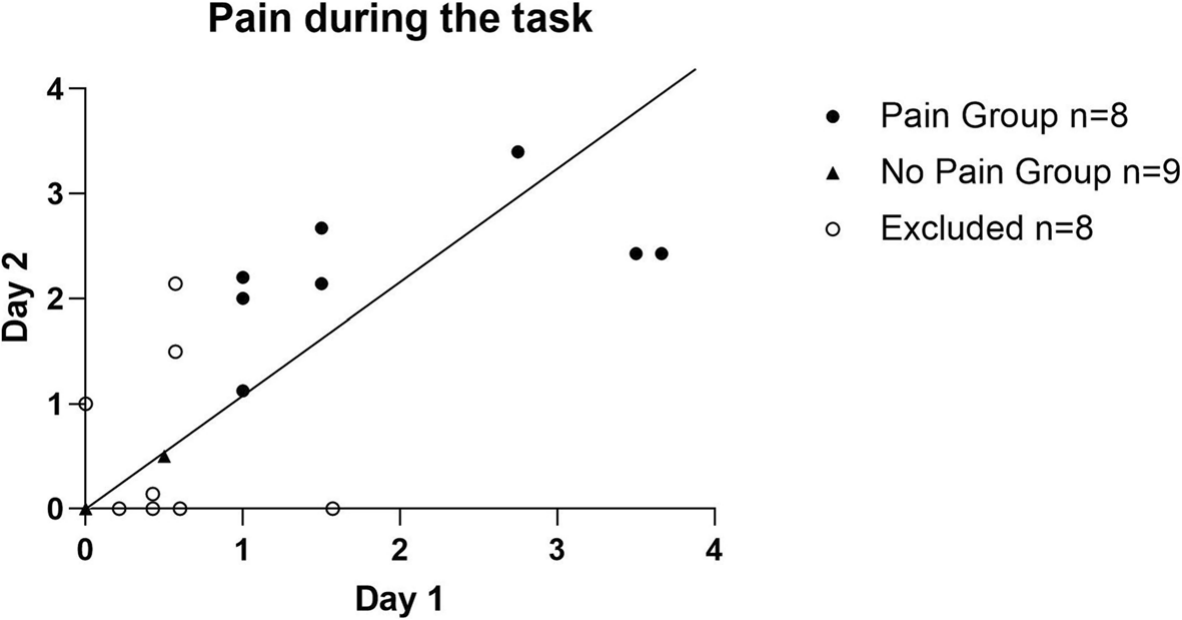
Pain during the task at Day 1 and Day 2. Data are presented as mean values of each time points pain was assessed during the task, for each participant. Only two No Pain group participants could be seen in Fig. 2 as they are all located at 0,0 except one participant

Of the 25 participants with ankle pathology who took part in this experiment, 8 (32%) participants experienced constant ankle/foot pain during the adaptation phase on both Day 1 and 2 (Pain group, mean pain level and standard deviation [sd]: Day 1 2.1 ± 1.3, Day 2 2.3 ± 0.8) and 9 (36%) participants did not experience pain during the experiment (No Pain group). Participants who reported intermittent pain (e.g., pain on Day 1 but not on Day 2) during the experiment were excluded from the analysis (*n* = 8 [32%]) as they could not be integrated either in the Pain group or in the No Pain group (Fig. 2). In the Pain group, 6 participants suffered from an ankle fracture, one from ankle osteoarthritis and one from talocalcaneal synostosis (mean number of days since injury or onset of pain = 167 ± 54 days). In the No Pain group, all the participants suffered from an ankle fracture (mean number of days since injury = 138 ± 33). Participants’ characteristics are described in Table 1. There was no statistical difference in terms of baseline characteristics (all *p* > .05) except for the BPI (*p* < .05). The mean level of pain experienced during the task in the Pain group was moderately stable between the days (ICC = .53). The mean level of pain was slightly superior on Day 2 (Fig. 2) but was not significantly different from Day 1 (Paired t-test *p* = .88).

**Table 1:** Characteristics of participants

	Pain group (***n*** = 8)	No Pain group (***n*** = 9)
**Gender** (male; female)	3; 5	3; 6
**Age** (X ± sd)	54.9 ± 13.9	43.6 ± 14.6
**Height, cm** (X ± sd)	164.4 ± 3.4	169.9 ± 8.7
**Mass, kg** (X ± sd)	71.0 ± 8.0	71.6 ± 4.4
**Number of days since injury**	167 ± 54	138 ± 33
**LEFS** (/80)	57.1 ± 14.2	67.7 ± 7.8
**Tegner score** (0-10)	2.6 ± 1.8	2.4 ± 1.7
**Brief Pain Inventory (Day 1)** (0-10)	2.0 ± 1.3*	0.7 ± 0.4*
**Brief Pain Inventory (Day 2)** (0-10)	1.5 ± 0.7	0.7 ± 0.4
**Dorsi-flexor strength (N)**	128.6 ± 34.9	151.9 ± 39.5
**Dorsi-flexion (°)**	25.8 ± 6.3	28.0 ± 6.7
**Pain during the task (Day 1)** (0-10)	2.0 ± 1.1	0
**Pain during the task (Day 2)** (0-10)	2.3 ± 0.7	0

X ± sd: mean and standard deviation. * *p* < 0.05

### Impact of clinical musculoskeletal pain on motor acquisition and retention

#### Motor learning performance

**Fig. 3:**
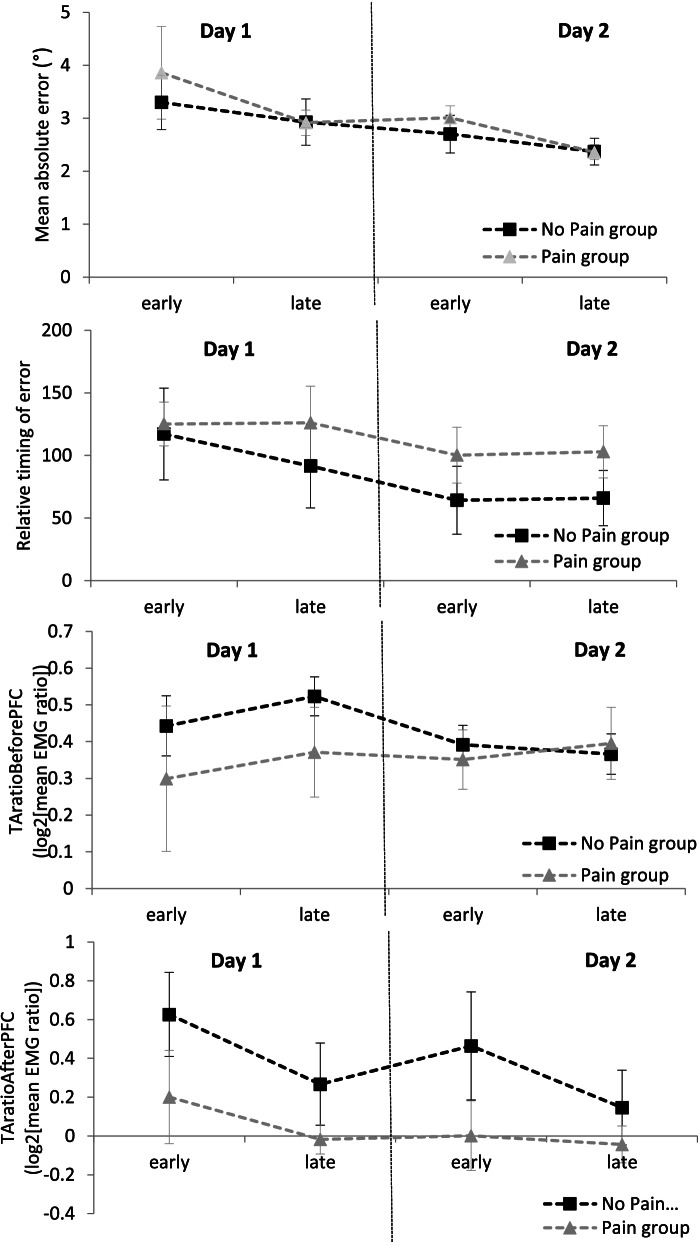
Pain vs No Pain groups results. Results are presented by mean ± SEM; TA: tibialis anterior, PFC: peak force command; Mean Absolute error = significant Day and Time effects were observed (*p* < .05 and *p* < .01, respectively). Relative timing of error = no main effect of either Day, Time or Group (all *p* > .05), and no interaction (all *p* > .05). TA_ratioBeforePFC_ = *Significant Group*Day interaction for TA_ratioBeforePFC_ (*p* = .014). TA_ratioAfterPFC_ = Significant Time effect for TA_ratioAfterPFC_ (*p* < .001)

A significant main effect of both Day and Time (*p* = .03; Relative treatment effect [RTE] Day 1 = .57, Day 2 = .43 and *p* = .005; RTE Early = .56 and Late = .44, respectively) was found on the Mean absolute error: both groups showed a decrease in Mean absolute error (i.e., improvement in global performance) between Day 1 and Day 2 and between Early and Late adaptation. No main effect of Group (*p* = .49; RTE Pain group = .54 and No Pain group = .47) or interaction (Group x Time: *p* = .29; RTE No Pain x Early = .52; No Pain x Late = .43; Pain x Early = .62 and Pain x Late = .46 / Group x Day: *p* = .80, RTE No Pain x Day 1 = .54; No Pain x Day 2 = .39; Pain x Day 1 = .60 and Pain x Day 2 = .48) was found (Fig. 3).

#### Motor learning strategy

No main effect of either Day (*p* = .08; RTE Day 1 = .56 and Day 2 = .44), Time (*p* = .88; RTE Early and Late = .50) or Group (*p* = .36; RTE No Pain group = .45 and Pain group = .55) and no interaction (Group x Time *p* = .76; RTE No Pain x Early = .46; No Pain x Late = .44; Pain x Early = .55 and Pain x Late = .56.44/ Group x Day *p* = .70; RTE No Pain x Day 1 = .53; No Pain x Day 2 = .37; Pain x Day 1 = .60 and Pain x Day 2 = .50) were observed on the Relative timing of error (Fig. 3).

A significant Group x Day interaction was observed on TA_ratioBeforePFC_ (*p* = .01; RTE No Pain x Day 1 = .64; No Pain x Day 2 = .50; Pain x Day 1 = .41 and Pain x Day 2 = .46). The No Pain group showed a decrease in TA_ratioBeforePFC_ between Day 1 and Day 2 (*p* = .04; RTE.64), while the Pain group did not show any change (*p* = .94; RTE .41). No main effect of Group (*p* = .30; RTE No pain group = .57 and Pain group = .44), Time (*p* = .47; RTE Early = .48 and Late = .52) or Day (*p* = .23; RTE Day 1 = .48 and Day 2 = .52) were found, and no other interaction (Group x Time *p* = .99; RTE No Pain x Early = .55; No Pain x Late = .59; Pain x Early = .42 and Pain x Late = .46) were observed (Fig. 3).

There was a significant Time effect (*p* < .001; RTE Early = .58 and Late = .42) for TA_ratioAfterPFC_: there was a decrease in TA_ratioAfterPFC_ between Early and Late adaptation for both groups. There was no Day (*p* = .26; RTE Day 1 = .54 and Day 2 = .46) or Group (*p* = .26; RTE No Pain group = .57 and Pain group = .44) effects and no interaction (Group x Time *p* = .60; RTE No Pain x Early = .66; No Pain x Late = .48; Pain x Early = .50 and Pain x Late = .37 / Group x Day *p* = .93; RTE No Pain x Day 1 = .61; No Pain x Day 2 = .54; Pain x Day 1 = .47 and Pain x Day 2 = .40) (Fig. 3).

## Discussion

### Feasibility of obtaining pain and no pain groups that are otherwise comparable

The first aim of this proof-of-concept study was to determine the feasibility to obtain two groups of participants with ankle pathology with or without residual pain during walking to investigate the effect of clinical pain on motor learning. We anticipated some challenges related to the study of musculoskeletal pain. As expected, some were identified, notably the fact that one third of participants could not be included in either the Pain or No Pain group due to the intermittent nature of their pain. It is hard to predict how painful a given task will be for the participants and to address this challenge in future studies, a larger sample size should be planned to account for the exclusion rate. Moreover, a minimal level of pain at inclusion should be considered as an inclusion criterion i.e., using an auto administered questionnaire, to attempt to minimize the exclusion rate.The BPI score has been shown to discriminate among musculoskeletal levels of condition severity, future studies should explore if a minimal BPI score could be used as an inclusion criterion to ensure the presence of pain during the task and determine the minimal BPI scores that correlate the presence of pain during locomotor tasks [37]. We also aimed to determine the feasibility to obtain two similar groups of participants with isolated ankle pathology in terms of baseline characteristics, because several factors other than pain may alter motor learning (i.e., time since the injury, stiffness, muscle atrophy, joint degeneration) [6, 38–40]., In the present study, we compared participants’ age, gender, height, mass, number of days since the injury, maximal dorsiflexion ROM and dorsiflexor maximal strength. We did not identify any significant difference between the two groups, which could be explained by the small sample size. However, our results suggests that it is feasible to obtain two similar musculoskeletal groups to investigate the effect of pain on motor learning while controlling for other variables.

### Impact of clinical musculoskeletal pain on motor acquisition and retention

The second objective of this study was to determine the influence of clinical musculoskeletal pain on motor learning performance and strategy. According to our results, musculoskeletal pain has no influence on global learning performance. Both groups showed motor learning by decreasing their Mean absolute error between the Early and Late stage and between Day 1 and Day 2. This is in accordance with similar results of two previous studies on experimental pain and locomotor learning [10, 15]. In both studies, and using the same gait adaptation task as in the current study, Bouffard et al. [10, 15] reported that both groups (i.e., participants receiving experimental pain vs no pain) showed similar Mean Absolute Error improvements over Time and Days. The few studies that have investigated motor learning and musculoskeletal pain also concluded that motor learning is preserved despite the presence of pain [11–13]. However, we can question whether motor learning in injured people is similar to that in healthy people, because looking at the Relative Treatment Effects (RTE), the lack of difference in motor learning between the two groups may be due to a lack of power. Indeed, small Main Effects were detected (Time effect RTE .44, *n* = 34 / Day effect RTE .43, *n* = 34), but small interactions were not (Group x Time RTE .43-.62, *n* = 18 and 16 / Group x Day RTE .39-.60, *n* = 18 and 16) [36]. In addition to the high exclusion rates, this supports the inclusion of a larger sample size when studying clinical population.

As for the learning strategy, it was different between the two groups. The No Pain group decreased their TA_ratioBeforePFC_ between Day 1 and Day 2, while the Pain group did not. However, this change in motor strategy was not supported by other variables such as the Relative timing of error, which limits the interpretation of this result. The lack of TA activity change before the PFC in the Pain group could be a protective motor behaviors related to pain anticipation [4] as increased antagonist corticospinal excitability have been observed during painful movement preparation [4]. Also, both groups showed a reduction of their mean TA_ratioAfterPFC_ between the Early and Late stage, but no Group effect or interaction were found. However, just like for the Mean Absolute Error, looking at Fig. 3 and at the RTEs, it is possible that the absence of Group effect and interaction are due to a lack of power.

Previous studies that have investigated locomotor learning task and experimental pain reported increased Relative timing of error in the presence of pain, reflecting a motor strategy that relies less on feedforward mechanisms, which did not occur in the present study [10, 15, 16]. The RTEs do not support the hypothesis of a lack of power for this variable (Relative Timing of Error RTE .45-.55, no effect) [36]. Experimental pain studies usually create a mean perceived level of pain of 5/10 [10, 15]. In the present study, the mean perceived level of pain was 2.2/10 in the Pain group during the task. If pain intensity influences the motor strategy, a lower intensity of musculoskeletal pain may have affected the magnitude of the effect measured in this study [6, 38–40]. The relationship between the pain subjective experience and the extent of interference with the motor system (measured for instance using transcranial magnetic stimulation) is much debated. On one side, no correlation has been observed between pain perception and pain-induced corticospinal excitability [7, 8, 41]. On the other side, when comparing the effect of controlled nociceptive stimulations, high intensity stimulations were found to result in more corticospinal inhibition than stimulations of lower intensity [5]. Interestingly, the later study showed potential influence of cognitive factors on these pain-motor system interactions, which could be an interesting avenue to explore in future studies.

### Strengths and limits

This proof-of-concept study is the first one to investigate the influence of pain on motor learning during a locomotor task in participants with musculoskeletal ankle condition. As expected, we did encounter some challenges related to the study of clinical musculoskeletal pain and made recommendations out of it. There was a limited sample size due to the high data exclusion rate (33%) and for some participants. These challenges limit the extrapolation of our results, but there are very few studies on clinical pain and motor learning, and none investigated locomotion. This study therefore has a unique contribution to the literature in addition to be useful to help further research to be conducted.

## Conclusion

From the results of this proof-of-concept study, musculoskeletal pain had no influence on motor learning performance but influenced the learning strategy used during the locomotor adaptation task, including a greater tibialis anterior activity before gait perturbation. Several barriers were identified for studying musculoskeletal pain, including the high rates of data exclusion because of some participants’ intermittent nature of pain, leading to a small sample size that might have affected the results. However, we were able to recruit two similar group of participants affected by isolate ankle pathology, with and without pain, and showed that it is feasible to investigate clinical pain and motor learning.

## Data Availability

The datasets generated and/or analysed during the current study are not publicly available due to ethical restrictions but are available from the corresponding author on reasonable request.

## Abbreviations

TA: Tibialis Anterior
MSKd: Musculoskeletal disorders
LEFS: Lower Extremity Functional Scale, an auto-administrated questionnaire
BPI: Brief Pain Inventory, an auto-administrated questionnaire
rAFO: robotized ankle foot orthosis, the custom-made robotized orthosis we used in this study
EMG: electromyographic
PFC: peak force command, the perturbation created with the rAFO while walking
TA_ratioBeforePFC_: Tibialis anterior EMG activity change before the perturbation
TA_ratioAfterPFC_: Tibialis anterior EMG activity change after the perturbation
RTE: relative treatment effect
ICC: intraclass correlation coefficient
SEM: standard errors of the mean

## References

[1] Nilsson G, Nyberg P Fau - Ekdahl C, Ekdahl C Fau - Eneroth M, et al. (2003). Performance after surgical treatment of patients with ankle fractures--14-month follow-up. Physiother Res Int.

[2] Nilsson G, Jonsson K, Ekdahl C (2007). Outcome and quality of life after surgically treated ankle fractures in patients 65 years or older. BMC Musculoskelet Disord.

[3] Simmonds MJ, Moseley GL, Vlaeyen JWS (2008). Pain, mind, and movement: an expanded updated, and integrated conceptualization. Clin J Pain.

[4] Neige C, Mavromatis N, Gagné M (2018). Effect of movement-related pain on behaviour and corticospinal excitability changes associated with arm movement preparation. J Physiol.

[5] Neige C, Brun C, Gagné M (2020). Do nociceptive stimulation intensity and temporal predictability influence pain-induced corticospinal excitability modulation?. NeuroImage.

[6] Mercier C, Léonard G (2011). Interactions between pain and the motor cortex: insights from research on phantom limb pain and complex regional pain syndrome. Physiother Can.

[7] Dubé JA, Mercier C (2011). Effect of pain and pain expectation on primary motor cortex excitability. Clin Neurophysiol.

[8] Billot M, Neige C, Gagné M (2018). Effect of cutaneous heat pain on corticospinal excitability of the tibialis anterior at rest and during submaximal contraction. Neural Plasticity.

[9] Clarke RW, Harris J (2004). The organization of motor responses to noxious stimuli. Brain Res Rev.

[10] Bouffard J, Salomoni SE, Mercier C (2018). Effect of experimental muscle pain on the acquisition and retention of locomotor adaptation: different motor strategies for a similar performance. J Neurophysiol.

[11] Parker RS, Lewis GN, Rice DA (2017). The association between Corticomotor excitability and motor skill learning in people with painful hand arthritis. Clin J Pain..

[12] Dagenais M, Brun C, Ohayon A (2021). Virtual reality in fibromyalgia: does altering visual feedback impact on pain and movement during reaching?. Front Virtual Real.

[13] Vittersø AD, Buckingham G, Ten Brink AF (2021). Characterising sensorimotor adaptation in Complex Regional Pain Syndrome. Cortex.

[14] Rittig-Rasmussen B, Kasch H, Fuglsang-Frederiksen A (2014). Effect of training on corticomotor excitability in clinical neck pain. Eur J Pain.

[15] Bouffard J, Bouyer LJ, Roy J-S (2016). Pain Induced during both the acquisition and retention phases of locomotor adaptation does not interfere with improvements in motor performance. Neural Plast.

[16] Bouffard J, Bouyer LJ, Roy J-S (2014). Tonic pain experienced during locomotor training impairs retention despite Normal performance during acquisition. J Neurosci.

[17] McPhail SM, Dunstan J, Canning J (2012). Life impact of ankle fractures: qualitative analysis of patient and clinician experiences. BMC Musculoskelet Disord.

[18] Bertrand-Charette M, Jeffrey-Gauthier R, Roy J-S (2021). Gait adaptation to a phase-specific nociceptive electrical stimulation applied at the ankle: a model to study musculoskeletal-like pain. Front Hum Neurosci.

[19] Wideman TH, Edwards RR, Finan PH (2016). Comparing the predictive value of task performance and task-specific sensitivity during physical function testing among people with knee osteoarthritis. J Orthop Sports Phys Ther.

[20] Kantak SS, Winstein CJ (2012). Learning–performance distinction and memory processes for motor skills: a focused review and perspective. Behav Brain Res.

[21] Spink MJ, Fotoohabadi MR, Menz HB (2010). Foot and ankle strength assessment using hand-held dynamometry: reliability and age-related differences. Gerontology.

[22] Sidaway B, Euloth T, Caron H (2012). Comparing the reliability of a trigonometric technique to goniometry and inclinometry in measuring ankle dorsiflexion. Gait Posture.

[23] Binkley J, Stratford P, Lott S (1999). The lower extremity functional scale (LEFS) scale development, measurement properties, and clinical application. Phys Ther.

[24] Dworkin RH, Turk DC, Wyrwich KW (2008). Interpreting the clinical importance of treatment outcomes in chronic pain clinical trials: IMMPACT recommendations. J Pain.

[25] Lapane KL, Quilliam BJ, Benson C (2014). One, two, or three? Constructs of the brief pain inventory among patients with non-cancer pain in the outpatient setting. J Pain Symptom Manag.

[26] Cleeland CS, Ryan KM (1994). Pain assessment: global use of the brief pain inventory. Ann Acad Med Singap.

[27] Panizzolo FA, Green DJ, Lloyd DG (2013). Soleus fascicle length changes are conserved between young and old adults at their preferred walking speed. Gait Posture.

[28] Dal U, Erdogan T, Resitoglu B (2010). Determination of preferred walking speed on treadmill may lead to high oxygen cost on treadmill walking. Gait Posture.

[29] Noël M, Cantin B, Lambert S (2008). An electrohydraulic actuated ankle foot orthosis to generate force fields and to test proprioceptive reflexes during human walking. IEEE Trans Neural Syst Rehabil Eng.

[30] Blanchette A, Lambert S, Richards CL (2011). Walking while resisting a perturbation: effects on ankle dorsiflexor activation during swing and potential for rehabilitation. Gait Posture.

[31] Noel M, Fortin K, Bouyer LJ (2009). Using an electrohydraulic ankle foot orthosis to study modifications in feedforward control during locomotor adaptation to force fields applied in stance. J Neuroeng Rehabil.

[32] Hermens HJ, Freriks B, Disselhorst-Klug C, Rau G, et al. Development of recommendations for SEMG sensors and sensor placement procedures. J Electromyogr Kinesiol. 2000. 10.1016/s1050-6411(00)00027-4. 10.1016/s1050-6411(00)00027-4 11018445

[33] Bagna M, Bouyer LJ (2010). A new approach for detecting and analyzing cutaneous reflexes during locomotion. J Neurophysiol.

[34] Koo TK, Li MY (2016). A guideline of selecting and reporting intraclass correlation coefficients for reliability research. J Chiropr Med.

[35] Noguchi K, Gel YR, Brunner E, et al. nparLD: an R software package for the nonparametric analysis of longitudinal data in factorial experiments. J Stat Softw. 2012;1(12). 10.18637/jss.v050.i12.

[36] Vargha A, Delaney HD (2000). A critique and improvement of the CL common language effect size statistics of McGraw and Wong. J Educ Behav Stat.

[37] Keller S, Bann CM, Dodd SL, et al. Validity of the brief pain inventory for use in documenting the outcomes of patients with noncancer pain. Clin J Pain. 2004;20. 10.1097/00002508-200409000-00005 15322437

[38] Massé-Alarie H, Flamand VH, Moffet H (2012). Corticomotor control of deep abdominal muscles in chronic low back pain and anticipatory postural adjustments. Exp Brain Res.

[39] Cowan SM, Hodges PW, Bennell KL (2002). Altered vastii recruitment when people with patellofemoral pain syndrome complete a postural task. Arch Phys Med Rehabil.

[40] Tsao H, Galea MP, Hodges PW (2008). Reorganization of the motor cortex is associated with postural control deficits in recurrent low back pain. Brain.

[41] Larsen DB, Graven-Nielsen T, Hirata RP (2018). Differential corticomotor excitability responses to hypertonic saline-induced muscle pain in forearm and hand muscles. Neural Plasticity.

